# Aesthetics of iris reconstruction with a custom-made artificial iris prosthesis

**DOI:** 10.1371/journal.pone.0237616

**Published:** 2020-08-13

**Authors:** Timur M. Yildirim, Ramin Khoramnia, Michael Masyk, Hyeck-Soo Son, Gerd U. Auffarth, Christian S. Mayer

**Affiliations:** 1 Department of Ophthalmology, University of Heidelberg, Heidelberg, Germany; 2 Department of Ophthalmology, Technical University of Munich, Munich, Germany; Nicolaus Copernicus University, POLAND

## Abstract

Patients with large iris defects not only suffer from functional disadvantages but also from aesthetic limitations. The aim of this study was to evaluate the aesthetic outcome of iris reconstruction using an artificial iris (AI). In this study, 82 eyes of 79 consecutive patients with mostly traumatic partial or total aniridia that underwent iris reconstruction surgery using a custom-made silicone AI (HumanOptics, Erlangen, Germany). Pre- and postoperative photographs of 66 patients were analysed subjectively and objectively. Subjective evaluation was based questionnaires. Objective evaluation included measurement of pupil centration and iris colour analysis. Averaged hues from iris areas were transferred to numerical values using the LAB-colour-system. Single parameters and overall difference value (ΔE) were compared between AI and remaining iris (RI), as well as AI and fellow eye iris (FI). Patients, eye doctors and laymen rated the overall aesthetic outcome with 8.9 ±1.4, 7.7 ±1.1 and 7.3 ±1.1 out of 10 points, respectively. Mean AI decentration was 0.35 ±0.24 mm. Better pupil centration correlated with a higher overall score for aesthetic outcome (p<0.05). The AI was on average 4.65 ±10 points brighter than RI and FI. Aniridia treatment using a custom-made artificial iris prosthesis offers a good aesthetic outcome. Pupil centration was a key factor that correlated with the amount of aesthetic satisfaction. The AI was on average slightly brighter than the RI and FI.

## Introduction

Eye contact plays an important role in successful social interaction. [1] In less than a second, often less than 120 ms, we unconsciously distinguish between sympathy and antipathy. [2–4] Minimal facial information is required as cues to judge credibility, competence and aggression in social interactions. [5] Altered eye appearance such as asymmetry or differences in colour, can be quickly noticed as annoying by an observer, and indeed the patient may perceive his or her perceive own appearance as bothersome (looking in a mirror or seeing self-photography). This critical behaviour, which is observable from early childhood, is considered genetically determined and based on evolutionary development. [6, 7] Aniridia, which is the partial or total absence of the iris is considered by the patient to be a severe aesthetic impairment, quite aside from visual symptoms it presents such as glare, reduced visual acuity and contrast sensitivity. [8]

Treatment of partial and total aniridia includes tinted anti-glare spectacles, coloured iris-print contact lenses, lamellar intrastromal corneal tattoos and iris suturing techniques. Although these approaches can improve functionality and some also enhance the aesthetic aspect, many patients find the treatment insufficient. [9–11]

Since 2007, a silicone-based iris prosthesis, individually hand-coloured based on patients’ iris photographs, the ArtificialIris® (HumanOptics, Erlangen, Germany), is available for intraocular iris reconstruction This implant has the potential to improve not only visual quality, but also the aesthetic appearance permanently. [12]

In current literature, there are only a few case reports and case series addressing the aesthetic outcome of this technique of iris reconstruction. [13–17] Our study aimed to systematically evaluate the aesthetic outcome of iris reconstruction using this custom-made silicone artificial iris prosthesis in a large group of patients with total and partial aniridia.

## Materials and methods

### Artificial iris

The Artificial Iris (AI) used in this study (ArtificialIris®, HumanOptics, Erlangen) is designed for posterior segment implantation in aphakic and pseudophakic eyes. The AI can be implanted alone or combined with an IOL. It has a 12.8 mm overall diameter with a fixed pupil size of 3.35 mm. Its thickness decreases from the pupillary margin (0.4 mm) to the periphery (0.25 mm). The AI is made from a biocompatible silicone material with a black posterior surface, which is optically opaque and coloured anterior surface that is based on a photograph of the patient’s residual iris tissue (RI) and the fellow eye’s iris (FI). Therefore, a high-resolution photograph needs to be obtained from both eyes, which is then printed and sent to the manufacturer for the implant to be individually hand-painted. By incorporating coloured silicone pigments in various layers, the manufacturer creates a three-dimensional plastic surface which resembles the natural iris surface with its crypts as well as giving a natural look that minimizes light reflections. [15] The Conformité Européenne certificate was granted to the ArtificialIris in 2011, and it was approved by the US Food and Drug Administration in the United States in 2018.

### Patient collective and image data

**Fig 1:**
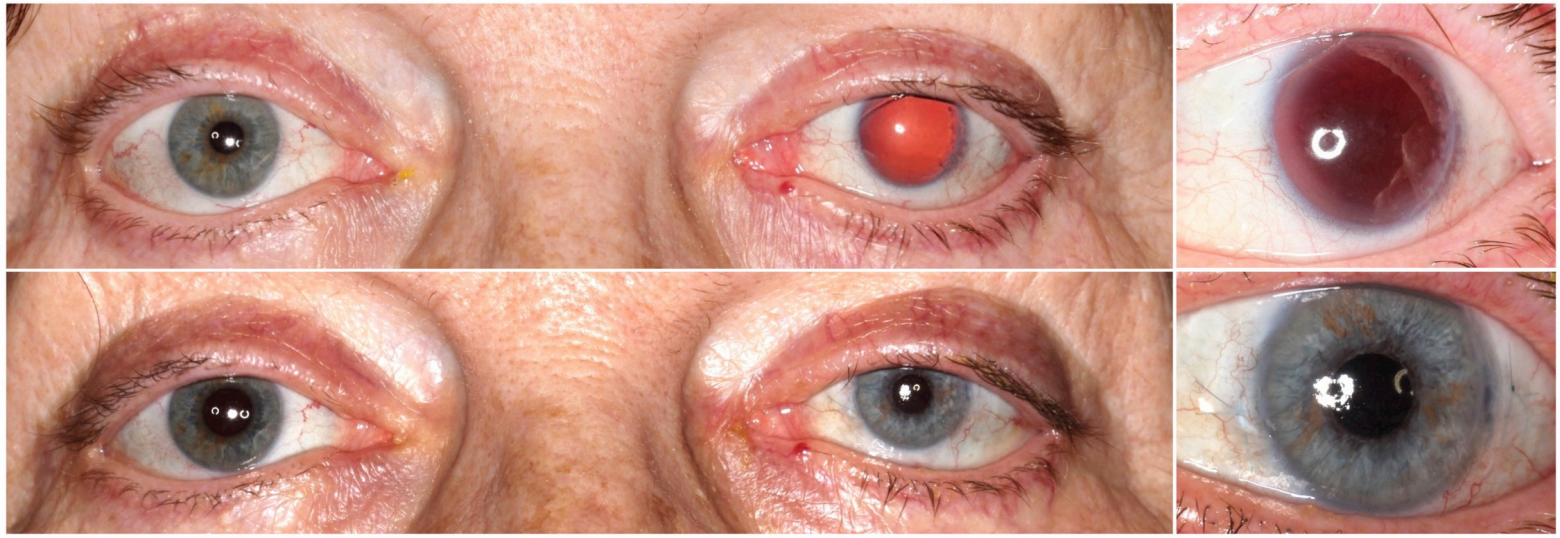
Subjective outcome evaluation. Binocular (left column) and close-up (right column) pre- and post-operative photographs for subjective outcome evaluation.

**Fig 2:**
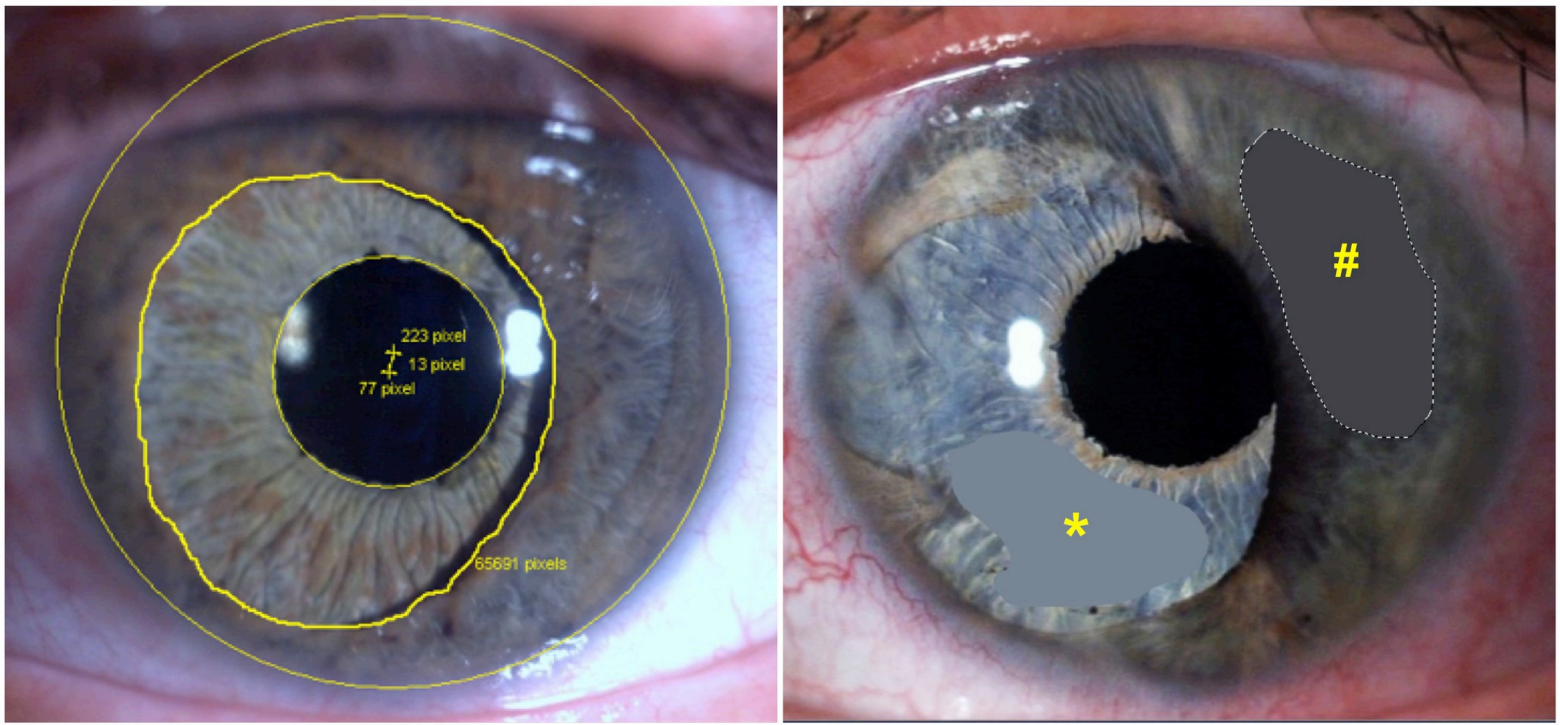
Methods for obtaining the objective outcome parameters. Lower part: Method for evaluation of pupil centration (left) and hue value (right). Left: Yellow lines mark the outer border of the cornea, the inner border of the residual iris tissue and the artificial pupil opening. The centres of the cornea and the artificial iris pupil are marked by a yellow +. The distance between the centres of the cornea and the artificial iris pupil are measured in number of image pixels and converted into millimetres. Right: Exemplary selections of residual iris tissue (yellow *#*) and artificial iris (yellow *) to average the iris hues that were then converted into a 3-digit value of the LAB-colour system.

**Fig 3:**
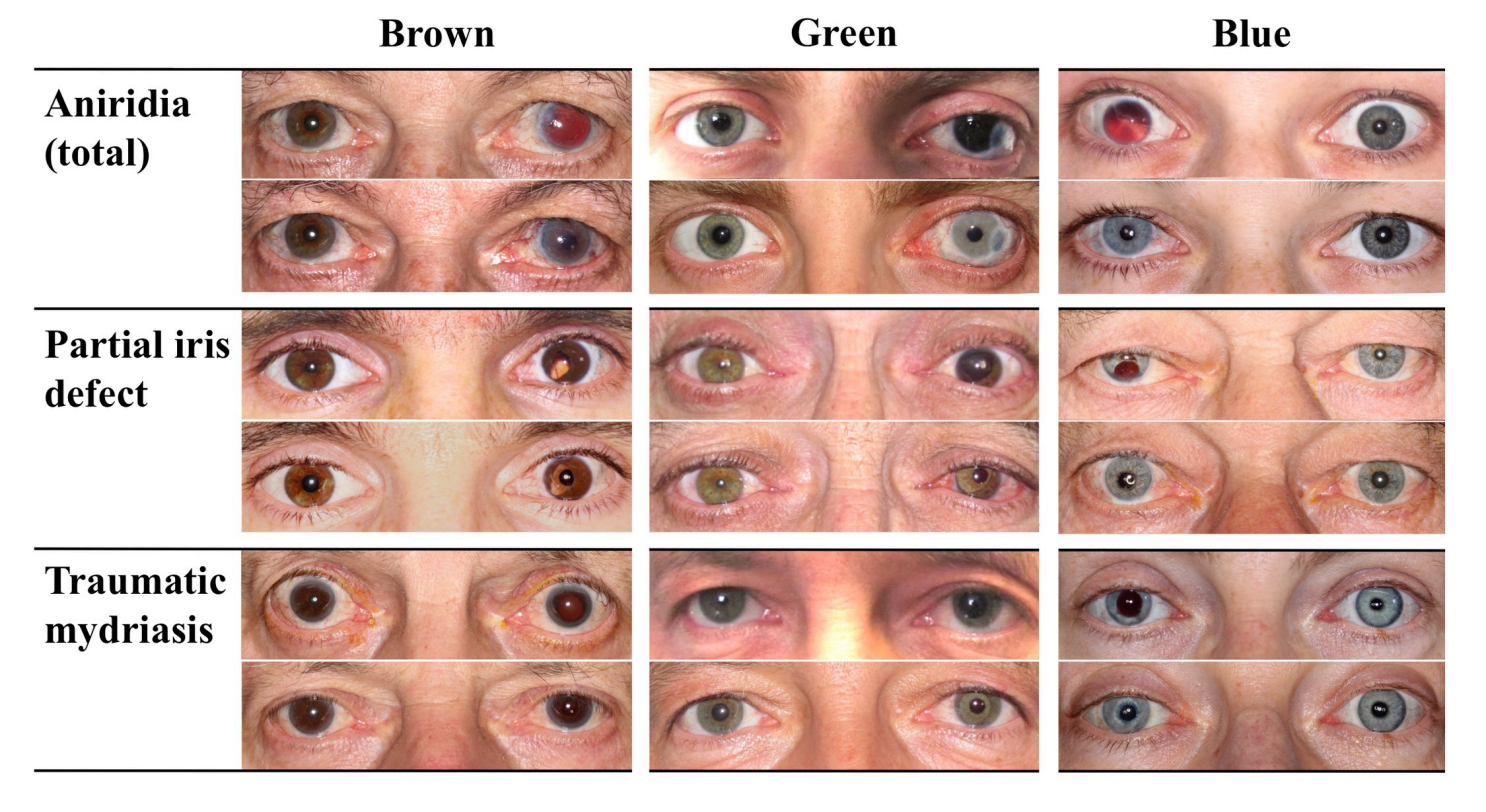
Collage of 9 sets of pre- and postoperative bilateral photographs of three different damage types and iris colours each. Damage types (aniridia, partial iris defect and traumatic mydriasis) are arranged horizontally and iris colours (blue, green and brown) vertically.

**Fig 4:**
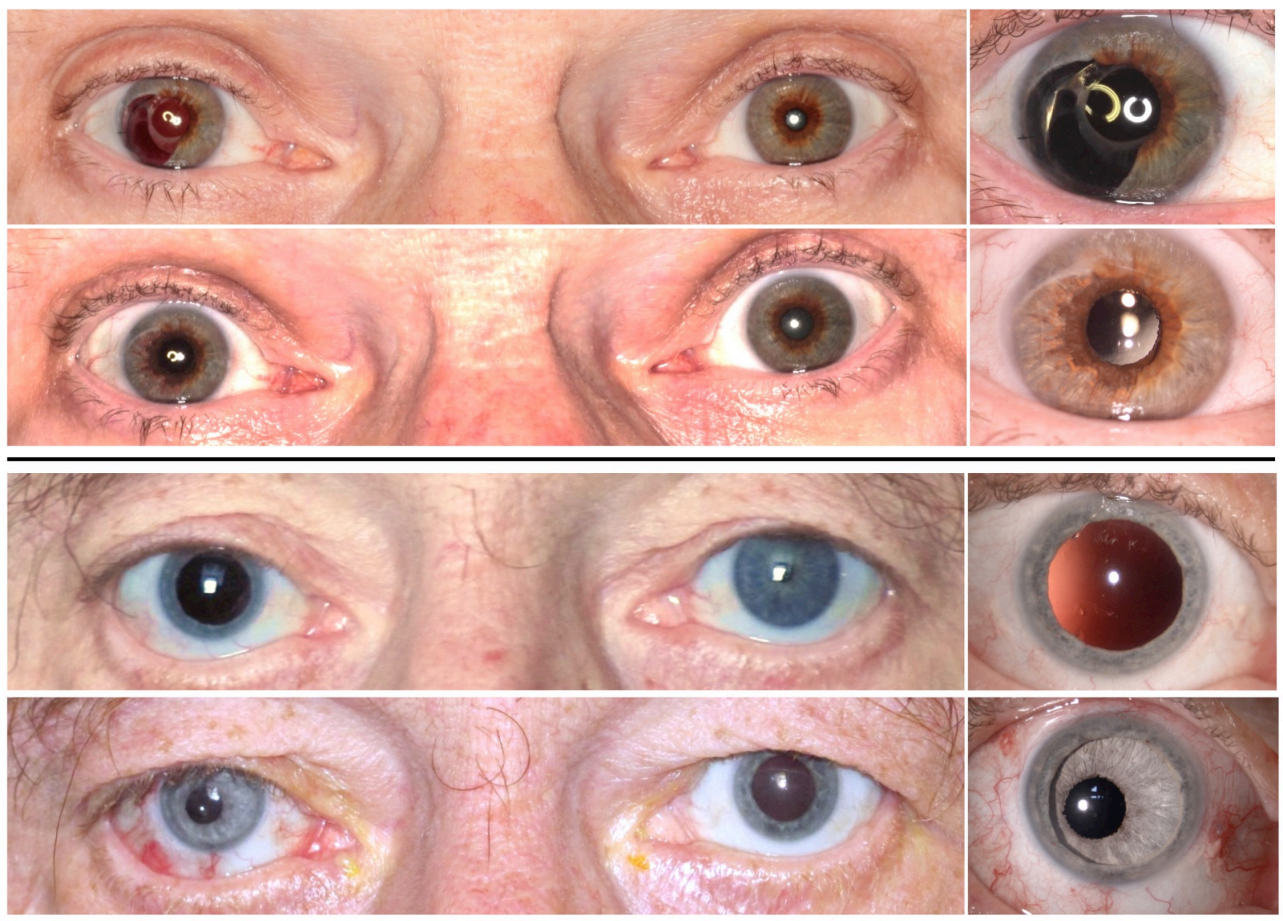
Objective pupil centration. Upper four images: Pre- and postoperative binocular (left) and close-up (right) photographs of the lowest postoperative artificial pupil decentration (0.02 mm). Lower four images: Pre- and post-operative binocular (left) and close-up (right) photographs of the highest post-operative artificial pupil decentration (1.04 mm).

**Fig 5:**
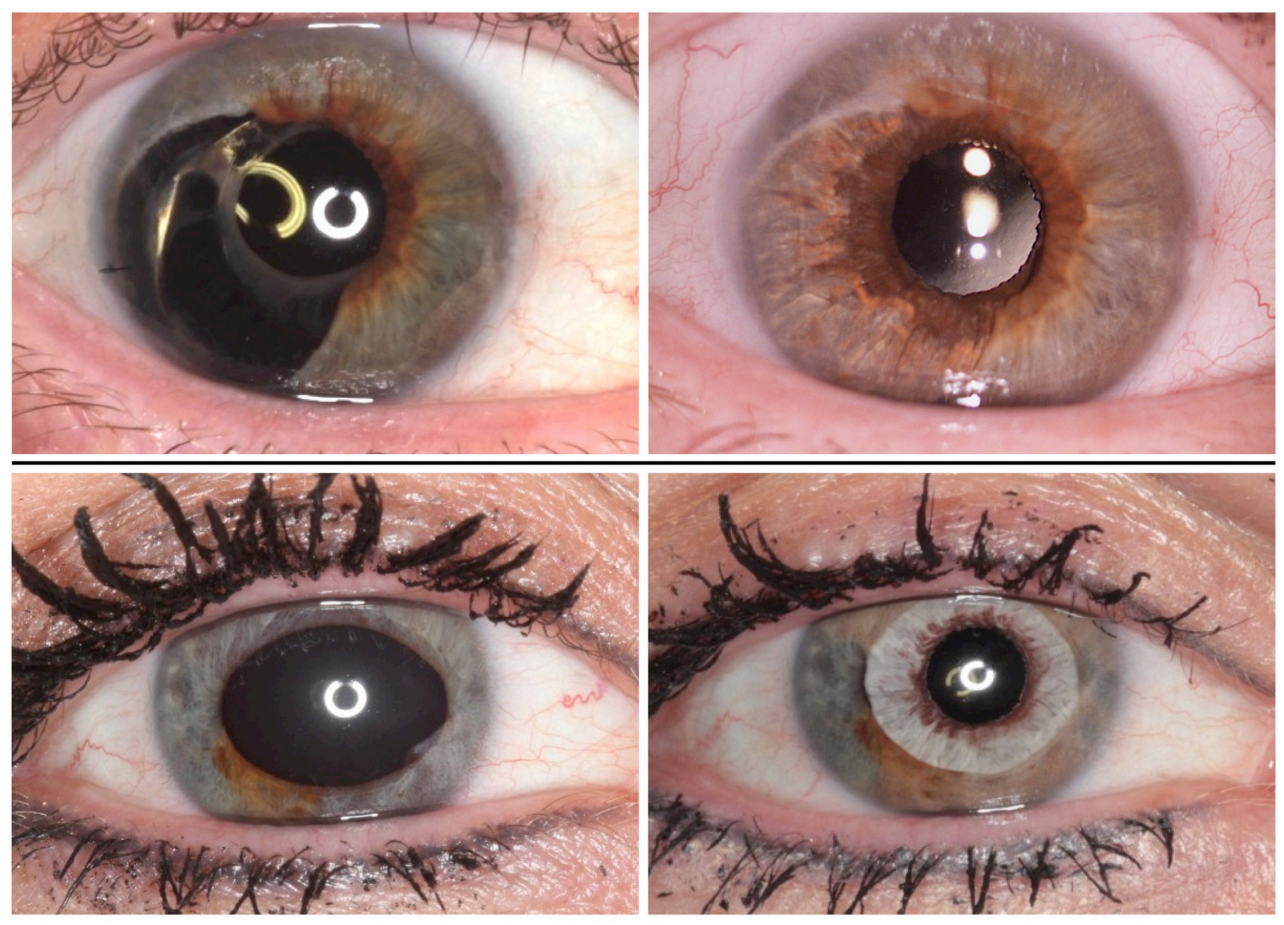
Objective colour differences between the artificial iris compared to the residual tissue. Pre- and post-operative photographs of the best, ΔE = 1.81 (upper row), and the worst, ΔE = 24.05 (lower row), colour match between artificial iris and residual iris tissue.

In this study, we analysed 82 eyes of 79 consecutive patients who, between 2011 and 2017, had surgery under general anaesthesia with implantation of complete artificial iris prosthesis. A single surgeon (CM) performed all surgeries. Pre- and postoperative photographs were obtained with a digital camera (EOS 600d, Canon, Tokyo, Japan) using standardized settings (100 mm macro lens, ring flash, automatic mode) and ambient light conditions. The study was approved by the ethics committee of the Technical University of Munich, Munich, Germany under the reference number 535/15 S and performed in accordance with the tenets of the Declaration of Helsinki. All participants gave written informed consent to participate in this study and patients concerned specifically consented to have potentially identifying images published in a scientific journal. Individuals from Figs 1–5 have given written informed consent (as outlined in PLOS consent form) to publish these photographs. We analysed data only where the patient completed the questionnaire and four standardized photographs (two pre- and two 3-months-postoperative) were available: one image of both eyes and a close-up image of the treated eye (Fig 1).

In 16 eyes, only an incomplete set of photographs could be obtained or one of the irises was not clearly recognizable or evaluable in the photographs (corneal opacity/ scar, reflection of camera flash).

Thus, 66 eyes, of 47 men and 19 women, with an average age of 55.6 ±16.2 (13–87) years met the inclusion criteria. In most of the cases the reason for aniridia was ocular trauma 61 (92.4%), with 36 (54.5%) blunt trauma and 25 (37.9%) perforating trauma. In 5 cases (7.6%) aniridia was congenital. Patients decided for surgery due to following reasons (multiple choices possible): 61 (92.4%) suffered from increased glare, 32 (48.5%) reduced visual quality, 25 (37.9%) aesthetic impairment, 2 (3%) diplopia and 1 (1.5%) light-dark disorder. Patients were divided according to their iris damage type into the three groups aniridia (total), partial iris defects, and traumatic mydriasis.

### Questionnaires

Subjective aesthetic outcome of all sets of photographs was evaluated using two versions of a questionnaire with visual analogue scales (VAS) from 1: poor to 10: excellent and grading scales from 1: very good to 6: very bad. One version was obtained from the treated patients, another version from eye doctors and laymen (Table 1).

**Table 1:** Questionnaire about the aesthetic outcome of the artificial iris surgery.

Question No.	Doctor’s and Layman’s questions (N = 50 and 30)	Patient’s questions (N = 70)
1	How do you rate the overall aesthetic outcome on a visual analogue scale (1: very bad, 10: excellent)?	
2	How do you rate the pupil centration of the AI (1: very good centration, 6: very bad centration)?	How has your quality of life changed after surgery on a visual analogue scale (1: negative, 5/6: unchanged, 10: very positive)?
3	How do you rate the AI hue difference compared to the RI (1: very good match, 6: very poor match)?	Would you repeat the treatment if you could decide again? (yes/ no)
4	How do you rate the AI hue difference compared to the FI (1: very good match, 6: very poor match)?	

All of the patients, 54 eye doctors and 30 laymen completed the questionnaire. The responses from 4 eye doctors had to be excluded due to formal mistakes. (Since the questionnaires for eye doctors and laymen were anonymous, these four could not be asked to redo their questionnaires).

### Objective image analysis

#### Pupil centration

Pupil centration was measured in the 3-months-postoperative close-up photograph using the Heidelberg Eye Explorer (Heyex, Heidelberg Engineering, Heidelberg, Germany). The distance between the geometric centre of the cornea and the centre of the artificial iris pupil was measured in number of image pixels and converted to millimetres (Fig 2).

#### Iris colour analysis

Image analysis was performed using an open source image processing software (GIMP 2.8 for IOS Mac). The largest possible areas of residual iris (RI), artificial iris (AI) and the iris of the fellow eye (FI) were selected manually using the area marker and colour was averaged (Fig 2). The colour was given as a 3-digit number according to the CIELAB (LAB)-colour-space.^A^

The LAB describes a three-dimensional colour space defined by the International Commission on Illumination (CIE) in 1976. It expresses colour as three values: L* for lightness from 0: lowest lightness (black) to 100: highest lightness (white), A* from green to red, and B* from blue to yellow. We specifically chose this colour system because unlike other systems like the RGB (red, green, and blue) or the CMYK (cyan, magenta, yellow, and key), the LAB-system is designed to approximate human vision and allows infinitely many possible colours.

To objectively evaluate the outcome regarding the hue match, mean AI values for L*, A* and B* were compared to the averaged values of the RI and FI. Furthermore, using a formula (the CIE2000 formula, http://zschuessler.github.io/DeltaE/learn/ (accessed on 14.10.2019)) a single difference value (ΔE) was be obtained from the 3-digit-LAB-number for each iris tissue pair; AI vs. RI, as well as AI vs. FI.^A^ In 10 out of 66 eyes, the AI vs. RI comparison could not be performed, as these 10 had no residual iris. Patients were divided into the three colour groups, blue, green and brown, and compared accordingly. The difference value (ΔE) is a measure for change in visual perception of two given colours. Delta E value ranges from 0 to 100. Sensation categories for this value are defined (in prints and graphics) as follows: not perceptible by human eyes (< 1); perceptible through close observation (1–2), perceptible at a glance (2–10), colours are more similar than opposite (11–49), colours are exact opposite (100).^A^

### Statistical analysis

Data was collected and analysed using Excel spread sheets (Excel 2011, Microsoft, Redmond, USA) and SPSS version 22.0 (SPSS Inc, Chicago, USA). Data was presented as arithmetic mean ±standard deviations (range) and number of patients (percentage). Nonparametric (Mann-Whitney-U-Test, Spearman's correlation) analyses were performed. A P value less than 0.05 was considered statistically significant.

## Results

Total aniridia was the reason for artificial iris implantation in 8 (12%) cases, partial iris defects in 34 (52%) cases and traumatic mydriasis in 24 (36%) cases (Fig 3).

### Overall aesthetic outcome

Overall evaluation of the aesthetic outcome (Question 1—VAS, 1–10) was 8.7 ±1.66 (2–10) in patients’ self-assessment. Results for overall rating were 7.73 ±1.07 (5.41–9.59) and 7.26 ±1.06 (3.93–9.23), respectively (p<0.05). The majority of patients, 61/66 (92.4%), would repeat the treatment if they could decide again (Question 3, patients’ questionnaire). The average change in patients’ quality of life (Question 2, VAS 1–10, patients’ questionnaire) was 7.99 ±1.85 (3–10). Exemplary, pre- and postoperative binocular photographs of the case with the best overall score of 9.54/10 (Question 1) are shown in Fig 1.

### Pupil centration

Mean pupil decentration was 0.35 ±0.24 (0.02–1.04) mm. Fig 4 shows two exemplary cases for different results for pupil centration.

Post-operative artificial pupil decentration for partial iris defects, total aniridia and traumatic mydriasis was 0.33 (±0.21), 0.31 (±0.26), 0.39 (±0.26), respectively, p>0.05. Eye doctors rated the pupil centration (Question 2—grades 1–6) better than laymen (2.05 ±0.66 vs. 2.35 ±0.70, p<0.05), Table 2.

**Table 2:** Summary of subjective and objective outcome measure for the aesthetic results.

Parameter	Objective parameters (pupil decentration in mm, hue difference in Δ E)	Subjective scores (eye doctors and laymen)			P Value
		Eye doctors and Laymen (N = 80)	Eye doctors (N = 50)	Laymen (N = 30)	
Question 1.—Overall Score (1–10)		7.55 (±1.01)	7.73 (±1.07)	7.26 (±1.06)	0.019*
Question 2.—Pupil centration	0.35 (±0.24)	2.16 (±0.66)	2.05 (±0.66)	2.35 (±0.70)	0.005*
Grades: 1–6					
Question 3.—Hue match (AI vs. RI)	10.21 (±5.39)	2.47 (±0.85)	2.47 (±0.91)	2.45 (±0.86)	0.974
Grades: 1–6					
Question 4.—Hue match (AI vs. FI)	11.79 (±7.33)	2.61 (±0.76)	2.58 (±0.85)	2.67 (±0.76)	0.464
Grades: 1–6					

Mean (±standard deviation), Score 1–10 (1: very bad to 10: excellent), Grades 1–6 (1 very good to 6 very bad)

*statistically significant difference between doctors and laymen (Mann-Whitney U test).

Objective pupil centration and subjective pupil centration score correlated statistically significant (Spearman's correlation, p<0.05), as did better pupil centration with an overall score (Spearman's correlation, p<0.05).

### Iris colour analysis

Mean lightness (L*)-value of the AI was on average 4.65 (±10) higher than the one of the RI and FI. A*- and B*- values only showed small mean differences between AI and RI and FI, with 1.25 (±7) and -0.02 (±9), respectively. Overall colour difference (Δ E) between AI and RI was slightly smaller than between AI an FI, with 10.21 (±5.39) and 11.79 (±7.39), respectively. Fig 5 shows the range of colour differences with the best and the worst objective outcomes.

Eyes with a blue iris showed a slightly higher hue difference value (ΔE) between AI and RI and AI and FI than eyes with green and brown iris colour, respectively (Table 3), without statistically significant difference, p>0.05.

**Table 3:** Hue differences in dependence of the iris colour. The difference value (ΔE*) was compared between the artificial iris and the remaining iris tissue, as well as the artificial iris and fellow eye iris.

Iris colour group	Number of eyes (percentage)	Hue difference value (ΔE*) between	
		AI and RI in mean (±SD)	AI and FI in mean (±SD)
Overall	66 (100%)	10.21 (±5.39)	11.79 (±7.33)
Blue	31 (47%)	11.65 (±5.36)	12.57 (±7.15)
Green	15 (23%)	8.94 (±4.42)	11.64 (±4.95)
Brown	20 (30%)	8.67 (±5.49)	10.69 (±8.81)

AI artificial iris, RI remaining iris, FI fellow eye iris

*The CIE2000 formula was used to obtain an overall difference value (ΔE) from a 3-digit-number from the LAB three-dimensional colour space defined by the International Commission on Illumination.

Doctors’ and Laymen’s scorings of the iris colour (Questions 3 and 4—grades 1–6) were similar and are summarized in Table 2. There was no statistically significant difference between eye doctors’ and laymen’s answers.

## Discussion

Aniridia treatment with implantation of a custom-made artificial silicone iris prosthesis offers good aesthetic results and high levels of patient satisfaction. It is important to emphasize that the primary goal of the surgery should always be to improve the patients’ visual impairment rather than enhancing the aesthetic appearance. Mayer et al. previously reported good functional results with this technique. [8, 18] In a prospective study, functional outcome of 37 consecutive cases of iris reconstruction using the AI were presented. Whereas best-corrected visual acuity did not change significantly, Pelli-Robson contrast sensitivity and impairment through glare improved three months after surgery. Even though the reason for surgery is to improve the quality of vision, the current analysis shows the positive side effect that the AI also enhances the aesthetic appearance of the patient. This ultimately leads to an improved quality of life.

We noted one crucial factor that influences a good aesthetic result: the better the centration of the artificial pupil, the better the overall aesthetic satisfaction rating from all questionnaire respondents. Even though mean objective pupillary decentration was as low as 0.35 mm, it had a significantly influence on the overall aesthetic result. As eye contact plays an important role in social interaction, this result seems to be reasonable and understandable. [1] A decentred pupil can lead to a squinting appearance as the angle between the optical axis and the centre of the pupil increases. Weissberg et al. aimed to identify the minimal angle of horizontal strabismus detectable by lay observers. [19] The authors found that even small angles are noticed: 70% of participants detected a 14.5Δ esotropia and 8Δ exotropia. [19] A more recent study from 2016 confirmed similar results with thresholds of 23.2Δ for esotropia and 13.5Δ for exotropia for a 70% positive detection rate. [20] Surgeons reconstructing the iris should therefore aim for good pupil centration to achieve the best possible aesthetic result.

The colour of the AI used in this study is customized to match the one of the residual and fellow iris. Overall, the scores for hue yielded good subjective grades even though several processing steps are required that have a potential to change the final AI’s colour. It is essential that the photographs obtained from the patients should be of high quality, acquired under the right light conditions, using standardized settings and a camera providing a sufficient quality of images. The printing process is also a potential source of error. One way to avoid this could be to send the reference photographs as digital files to the manufacturer. In this study, beside of subjective evaluation, we applied an objective method to evaluate the colour outcome. To the best of our knowledge, as no comparable study has been conducted before, we decided to utilize an objective method using the LAB-colour-system to determine differences between AI and RI and AI and FI, respectively. The CIELAB colour space is typically used for graphics and prints. Objective hue evaluation 3 months postoperatively showed that the AI colour was on average slightly brighter than the patients’ own tissue (FI and RI). One example of this finding can be nicely visualized in Fig 5 (lower row), showing the worst result regarding the overall colour difference value (Δ E). In this case, the postoperative AI colour is clearly brighter than the patient’s tissue. A possible explanation would be that the AI looks brighter under water than when dry. The different surface relief structure of the AI compared to the real iris could lead to increased light reflection which makes it look brighter under direct illumination within the eye. Rickmann et al. reported in 2016 long-term clinical outcome in 34 patients that were treated with a custom-made, flexible artificial iris prosthesis. They found in almost one fourth of the cases (23.5%), that the remaining iris tissue darkened during the post-implantation period, in a follow-up of at least 2 years. [21] This finding from Rickmann et al. together with our data suggests that one might choose a slightly darker AI in order to compensate for its slightly brighter appearance within the eye and to anticipate the expected darkening of the patient’s remaining iris tissue. Our long time surveillance of the patients revealed instead of changing colour of the remnant iris, a residual iris retraction syndrome (RITS). [22] Regarding the A*- and B*- values, average differences were very small suggesting that the hand-painting manufacturing process provides reliable colour match.

An interesting outcome of the presented data is, that mean scores for the same questions differed between the groups. Overall score and pupil centration was rated significantly better by eye doctors than laymen, hue matches did not change between the groups. One possible explanation could be that eye doctors have a better understanding of the difficulties of such surgeries and more appreciate the results since fixation of the AI is often complicated. The AI may be placed in the ciliary sulcus without sutures in the case of a pre-existing intracapsular intraocular lens, or it is implanted in the capsular bag together with a new intraocular lens, or it is sutured to the sclera with or without an attached intraocular lens. [23] Therefore the surgeon encounters various difficulties that are sometimes hard to predict. The difference in this understanding might explain the slightly better scoring of the eye doctors. Nevertheless, overall scoring was very good in both of the groups, with 7.73/10 and 7.26/10 for eye doctors and laymen, respectively.

Regardless of the good aesthetic results of the artificial iris prosthesis, results should be interpreted with caution and not lead to the conclusion that the implant should be considered as a device for cosmetic enhancement. As previously shown, short and long-term safety parameters have to be taken into account. [8, 24] In a previous study on 37 eyes, IOP did not elevate in a statistically significant way 3 months after surgery, but even in experienced hands, there was a small but statistically significant endothelial cell count loss of about 5% 3 months after surgery. [8] In a further study by the same group, authors analysed complications of this surgery. [24] In a group of 51 patients treated with the AI, about one third (25.5%) suffered from complications from which the majority 11/13 were classified as severe (requiring any surgical intervention). The authors found that most of the complications occurred during the first surgeries of the single surgeon in this study and the complication rate dropped from 83.3% to 0% within the first three years. [24] This suggests that the AI should only be used by experienced surgeons, especially in cases where not only functional, but also aesthetic reasons are considered in the decision-making for surgery. In our study, the use the artificial iris prosthesis was always based on a case-by-case decision, taking into account several different aspects including not only the type of iris damage but also the eye’s functional potential and the patient’s preference after thorough education about all possible options. In some of the cases, other techniques of iris reconstruction such as iris suturing or a pupil cerclage could have been alternative treatment options. It is important to note that the ArtificialIris should also not be confused with solely cosmetic implants, e.g. NewIris^®^ (Kahn Medical Devices Corp, Panama City, Panama) or others, that serve also as cosmetic implants. There are several reports that have shown that those devices can lead to severe complications. [14, 25, 26]

Our study certainly has some limitations: First, no validated questionnaire suited answering the current research question and therefore, as part of this study, a non-validated questionnaire was used. Furthermore, the inclusion of different damage groups limits general conclusions about this technique. The presented data does not allow conclusions about functional and anatomical aspects, which have been presented elsewhere. [8, 21, 23, 24]

However, this study revealed several interesting aspects about the aesthetic result of iris reconstruction using the presented custom-made artificial iris prosthesis. In conclusion, this technique offers an overall good subjective aesthetic result in addition to the formerly reported functional benefits. The hue was slightly brighter than required, suggesting that one should aim for a slightly darker colour. Finally, pupil centration was found to be a major factor contributing to the level of aesthetic success.
